# Occurrence of virulence gene signatures associated with diarrhoeagenic and non-diarrhoeagenic pathovars of *Escherichia coli* isolates from some selected rivers in South-Western Nigeria

**DOI:** 10.1186/s12866-015-0540-3

**Published:** 2015-10-08

**Authors:** Yinka Titilawo, Larry Obi, Anthony Okoh

**Affiliations:** SAMRC Microbial Water Quality Monitoring Centre, University of Fort Hare, Alice, 5700 South Africa; Applied and Environmental Microbiology Research Group, Department of Biochemistry and Microbiology, University of Fort Hare, Alice, 5700 South Africa

**Keywords:** Prevalence, *Escherichia coli* pathovars, Virulence genes, Diarrhoeagenic, Surface water, Nigeria

## Abstract

**Background:**

Diarrhoeal diseases are attributable to unsafe water stemming from improper sanitation and hygiene and are reportedly responsible for extensive morbidity and mortality particularly among children in developed and developing countries.

**Methods:**

Water samples from selected rivers in Osun State, South-Western Nigeria were collected and analyzed using standard procedures. Escherichia coli isolates (n=300) were screened for 10 virulence genes using polymerase chain reaction for pathotyping.

**Results:**

While the virulence gene (VG) *lt* for enterotoxigenic *E. coli* had the highest prevalence of 45 %, the enteropathogenic *E. coli* genes *eae* and *bfp* were detected in 6 and 4 % of the isolates respectively. The VGs *stx*1 and *stx*2 specific for the enterohemorrhagic *E. coli* pathotypes were detected in 7 and 1 % of the isolates respectively. Also, the VG *eagg* harboured by enteroaggregative pathotype and diffusely-adherent *E. coli* VG *daa*E were detected in 2 and 4 % of the isolates respectively and enteroinvasive *E. coli* VG *ipa*H was not detected. In addition, the VGs *pap*C for uropathogenic and *ibe*A for neonatal meningitis were frequently detected in 19 and 3 % of isolates respectively.

**Conclusions:**

These findings reveal the presence of diarrhoeagenic and non-diarrhoeagenic *E. coli* in the selected rivers and a potential public health risk as the rivers are important resources for domestic, recreational and livelihood usage by their host communities.

## Background

Globally, diarrhoeal diseases and other related gastrointestinal illnesses constitute one of the most important causes of illness and death in the world particularly among infants and young children [1–3], with most of such illnesses contracted through ingestion of polluted waters. Ascertaining the qualities of fresh and marine waters relies heavily on the use of *Escherichia coli* and *Enterococcus* spp. commonly found in mammalian faeces [4, 5]. *Escherichia coli* is the most abundant facultative anaerobe. Most are commensals in the human intestinal microflora, but certain strains have virulence properties that may account for life-threatening infections. The pathogenicity of a particular *E. coli* strain is primarily determined by specific virulence factors which include adhesins, invasins, haemolysins, toxins, effacement factors, cytotoxic necrotic factors and capsules [6, 7], and these have been implicated in human and animal diseases worldwide with the pathogenic strains being categorized into intestinal pathogenic *E. coli* (InPEC) and extra-intestinal pathogenic *E. coli* (ExPEC) on the basis of their virulence factors and clinical symptoms [8, 9]. InPEC can be further classified into enterotoxigenic *E. coli* (ETEC), enteropathogenic *E. coli* (EPEC), enterohemorrhagic *E. coli* (EHEC), enteroinvasive *E. coli* (EIEC), enteroaggregative *E. coli* (EAEC) and diffusely adherent *E. coli* (DAEC) [9–11], and ExPEC into uropathogenic *E. coli* (UPEC), neonatal meningitis *E. coli* (NMEC) and avian pathogenic *E. coli* (APEC). Other diarrhoeagenic *E. coli* pathotypes have been proposed, such as cell-detaching *E. coli* (CDEC); however their significance remain uncertain [2, 12].

Common reservoirs of ETEC and EPEC include humans, ruminants, porcine, other domesticated animals such as goats, dogs and cats [10, 13, 14]. EHEC have been isolated from various ruminants, primarily cattle [15]. The principal reservoir for EIEC, EAEC and DAEC are humans [9, 13]. While UPEC and NMEC are commonly isolated from humans, APEC have been attributed to avian infections from poultry [9, 16]. Enterotoxigenic *E. coli* (ETEC) have been found associated with infantile and traveler’s diarrhoea; EPEC with acute infantile diarrhoea; EHEC with sporadic outbreaks of haemorrhagic colitis and hemolytic-uremic syndrome in humans; EAEC with persistent gastroenteritis and diarrhoea in infants and children and is prevalent in developing countries; and EIEC produces shigellosis-like diseases in children and adults, with invasive intestinal infections, watery diarrhoea, and dysentery in humans and animals [9, 10]. DAEC strains have also been associated with diarrhoeal disease in different geographic areas [17]. Uropathogenic *E. coli* (UPEC) enters the urinary tract and travels to the bladder to cause cystitis and, if left untreated, can ascend further into the kidneys to cause pyelonephritis. Septicaemia can occur with both UPEC and neonatal meningitis NMEC, and NMEC can cross the blood–brain barrier into the central nervous system, causing meningitis [18].

Contamination of surface waters with pathogenic strains of *E. coli* has been implicated in increasing number of disease outbreaks and deaths [19, 20] Disease outbreaks related to exposure to contaminated freshwaters are well documented [20–23]. The occurrence of pathogenic *E. coli* strains harbouring virulence genes (VGs) in environmental waters could be linked to contamination by storm events, faeces from domestic and wild animals as well as humans, runoffs from agricultural lands, sewage overflows, farm animals, pets and birds [11, 24–27]. However, only a few studies have investigated the presence of *E. coli* strains carrying VGs in environmental waters [28–34]. Exposure to recreational waters has been linked to high numbers (21 out of 31) of reported *E. coli* O157:H7 disease outbreaks in the United States from 1982 to 2002 [35]. Prevalence studies on the various *E. coli* pathotypes are important since it has been shown through various studies that the prevalence of diarrhoeagenic *E. coli* is region-specific [36]. Studies on the prevalence of DEC categories and their importance in diarrhoea have not been carried out extensively in Nigeria [37–41], with investigations on the southwestern axis being scantily documented on stool samples but not on environmental waters. A controlled study using the traditional culture/serology technique and polymerase chain reaction (PCR) was designed to ascertain the level and spectrum of bacterial pathogens and define the association of various categories of *E. coli* with diarrhoea in Enugu and Onitsha, Southeastern Nigeria [42]. To the best of our knowledge, no investigation on *Escherichia coli* pathotypes distribution has been carried out on the freshwater environments of Nigeria. Hence, in this paper, we report for the first time the prevalence and distribution of diarrhoeagenic *E. coli* pathotypes in surface waters in Osun State, South-Western Nigeria.

## Results

### *E. coli* confirmation

Of the 480 presumptive *E. coli* isolates recovered from the sampling sites, 410 were confirmed to be *E. coli* out of which 300 isolates made up of 30 from each sampling location were pooled together for further analysis. Figure 2 below shows the gel electrophoresis picture of the PCR products of the *uid*A gene amplification.

**Fig. 1:**
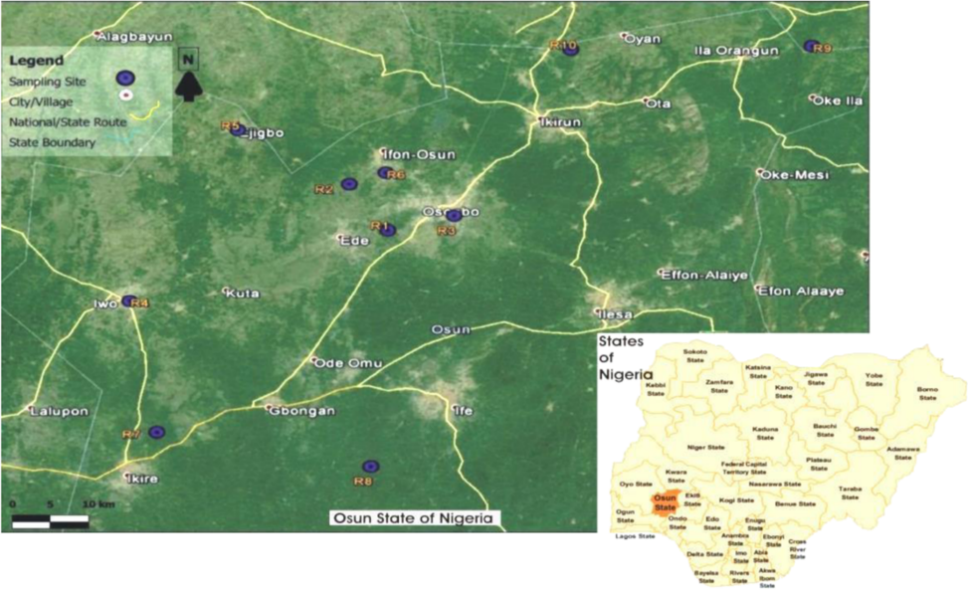
Map of Osun State Nigeria showing the locations of the sampling sites. Reprinted from [98] Sci Total Environ, 523, Titilawo Y., Obi L. and Okoh A., Antimicrobial resistance determinants of *Escherichia coli* isolates recovered from some rivers in Osun State, South-Western Nigeria: Implications for public health, 82–94, Copyright (2015), with permission from Elsevier

**Fig. 2:**
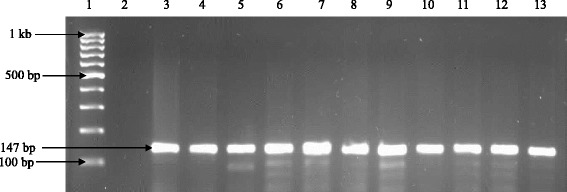
PCR products of the amplification of *uid*A gene. Lane 1: molecular weight marker (100 bp); lane 2: negative control; lane 3: positive control (ATCC *25922*); lanes 4–13: positive isolates

### Prevalence of *E. coli* in the river samples

**Fig. 3:**
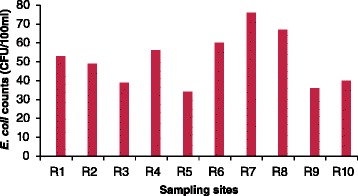
Comparative mean counts of *E. coli* at the ten sampling sites

Confirmed *E. coli* isolates in the river samples at all sites ranged between 34 CFU/100 ml at the R5 site and 76 CFU/100 ml at the R7 site (Fig. 3).

### Prevalence of virulence genes (VGs) amongst confirmed *E. coli* isolates

**Fig. 4:**
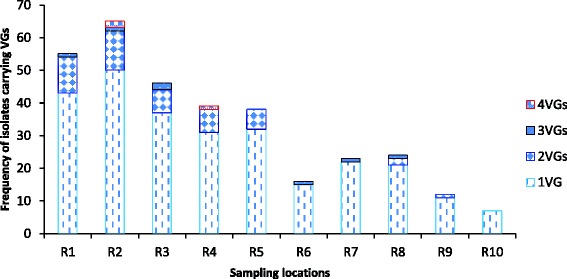
Comparative distribution of the virulence genes (VGs) in *E. coli* isolates

**Fig. 5:**
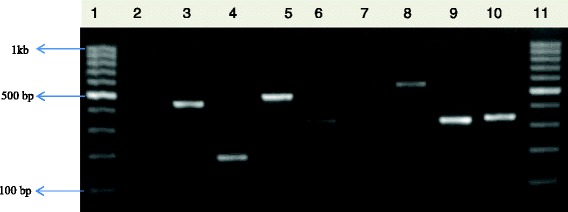
A representative gel electrophoresis profile of different virulence genes of the diarrhoeagenic and non-diarrhoeagenic *E. coli* isolates. Lane 1: molecular weight marker (Thermo Scientific 100 bp DNA ladder), lane 2: negative control, lane 3: *lt*, lane 4: *eagg*, lane 5: *eae*, lane 6: *stx*1, lane 7: *stx*2 and lane 8: *daa*E

Among the 300 confirmed *E. coli* isolates assessed for the various VGs, 273 (91 %) harboured at least 1 VG while 27 (9 %) isolates harboured none. Overall, 91 % of the isolates were found to harbour between 1 and 4 VGs (Fig. 4). The modal occurrence of 1 VG was recorded at site R2 with 50 (33 %) of the isolates being positive for 1 VG each. The prevalence of multiple VGs in the *E. coli* isolates was equally higher at R2 than at other sites. The heat-labile toxin, *lt* gene was the most commonly detected gene at site R1 in 24 (80 %) of the isolates, followed by the adhesion *pap*C gene, detected in 17 (57 %) of the isolates (Fig. 4). Similarly, the modal occurrence of 2 VGs was at R2 in 12 (10 %) isolates. None of the isolates from R6, R7 and R10 sites harboured 2 VGs Also, the modal prevalence of 3 VGs and 4 VGs were at R3 and R2 each in 2 (7 %) (Fig. 4). The invasion plasmid antigen gene, *ipa*H was not observed throughout the study and was therefore omitted from subsequent analysis. The representative gel electrophoresis profiles of amplified products of the investigated diarrhoeagenic and non-diarrhoeagenic coding genes are shown in Fig. 5.

Statistical analysis using one-way ANOVA was performed on the pooled data in order to further explore the distribution of the remaining 9 VGs among all the ten sites. There was a significantly higher occurrence (*P* < 0.05) of 1 VG at sites R4, R5, R7 and R8 than at any other sites. While sites R1 and R2 were not significantly different in the prevalence of 2 VGs in their isolates (*P* > 0.05), a significant difference was observed when compared to the other 8 sites tested (*P* < 0.05). Similarly, the differences in the occurrences of 3 VGs among sites R1, R2, R6, R7 and R8 were not significant (*P* > 0.05).

### Comparative prevalence of *E. coli* pathotypes

**Fig. 6:**
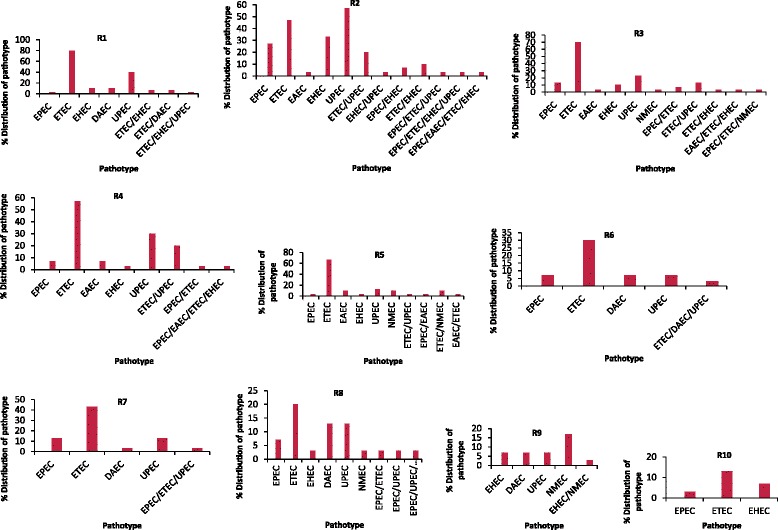
Comparative distribution of *E. coli* pathotypes from all ten sites in Osun State, South-Western Nigeria

To identify the prevalence of different pathotypes of *E. coli* isolates in all the ten sites, VGs were grouped according to their association with different *E. coli* pathotypes. The percentage site-specific distribution of the *E. coli* pathotypes in the ten sampling locations is shown in Fig. 6. Overall, isolates belonging to the intestinal ETEC pathotype were the most commonly detected (45 %), followed by the extra-intestinal UPEC (19 %) and the lowest was EAEC (2 %). Approximately, 21, 18 and 12 % of the *E. coli* isolates in sites R3, R5 and R8 could be placed into five main pathotypes with EPEC, ETEC, EHEC, UPEC and NMEC mostly observed. While EAEC was noticed at sites R3 and R5, DAEC was detected at R8. Similarly, the ETEC pathotype was commonly found associated with UPEC (20 %) at sites R2 and R4 each, and 13 % at site R3, followed by ETEC/EHEC and ETEC/NMEC (10 %) each (Fig. 6).

In addition, the percentage distribution of pathotypes was uniform at almost all the sampled sites except R6, R7 and R10. Approximately, 3 % of *E. coli* isolates could be differentiated into more than three pathotypes and this was observed at sites R1, R2, R3, R6, R7 and R8 in the same proportion (3 %) each, with the ETEC/EPEC/UPEC pathotypes commonly found at R2 and R7. Conversely, 3 % of the isolates positive for multiple VGs could only be grouped into four defined pathotypes as observed at sites R2 and R4 only, with ETEC/EPEC/EHEC/EAEC common to both (Fig. 6). Other sampling sites’ isolates did not carry multiple VGs sufficient enough to be categorized into four pathotypes.

### Comparison of *E. coli* VG profiles from ten sites

**Fig. 7:**
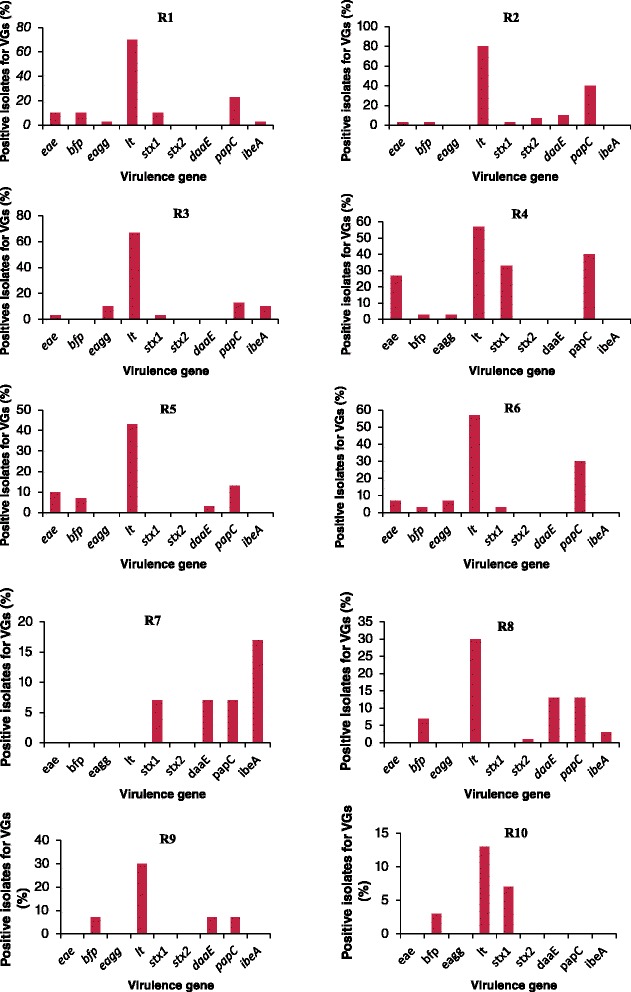
Comparative distribution of VGs in *E. coli* isolates from all ten sites in Osun State, South-Western Nigeria

VGs were further classified into toxin, adhesion and invasion genes based on functional characteristics of the genes (Table 2). This enabled us to identify the prevalence of different virulence genes of *E. coli* with observable differences in each site. A comparative analysis of the distribution of the 9 VGs observed across all the ten sites is presented in Fig. 7. In general, the highest frequency of VGs in *E. coli* isolates was obtained at site R2 (16 %), followed by site R1 (15 %) and the lowest at site R10 (2 %). Overall, the highest occurrences of VGs were observed at R1 and R3, with 7 VGs each and the lowest at site R10 with 3 VGs. The VGs *pap*C and *lt* were the most frequently detected across all the sites, whereas the *bfp*, *eagg*, *stx*2 and *ibe*A genes were infrequently detected (Fig. 7). Among the toxin genes screened in this study, *lt* was the most prevalent gene at all sites (45 %) except at site R9 where it was not detected. Likewise, *pap*C was the most commonly detected adhesion gene observed across all the sampling sites (19 %) with *eagg* gene being the least (2 %). Conversely, of the two invasion genes assessed, *ibe*A was detected at a very low prevalence (3 %) while *ipa*H was not detected in all the isolates (0 %) (Fig. 7).

A comparison between the sites was made (ANOVA) to determine if the sites were similar or different on the basis of occurrence of VGs. Both the sites R1 and R2 were significantly different (*P* < 0.05) in the occurrence of *stx*1 toxin and *eae* adhesion genes compared to other sites, whereas the difference between the occurrence of *stx*2 toxin gene in the *E. coli* isolates from site R2 was found to be statistically non-significant (*P* > 0.05) in relation to those from the other sites except site R1. Similarly, there was a significant difference in the occurrence of *ibe*A invasion gene among the isolates obtained at sites R5 and R9 (*P* < 0.05).

## Discussion

The present study investigated the distribution and frequency of defined pathotypes of *E. coli* isolates in river water samples from Osun State, South-western Nigeria. Generally, the mean annual counts of the presumptive *E. coli* obtained in all the sampling sites were relatively high. *E. coli* has been used extensively as one of the major faecal indicator bacteria due to the previous notion that it has limited survival ability in the environment though recent studies have suggested that some pedigrees of *E. coli* have adapted and acclimatized within tropical, subtropical and even temperate regions [43, 44], and as such could be the germane reason for *E. coli* to flourish especially within freshwaters. In this study, sites R1, R2, R4, R6 and R7 with higher counts of *E. coli* were those located in pasture and peri-urban catchments with multiple sources of faecal pollution such as run-offs and dungs from cattle, horses and wild animals. There is a high likelihood that the isolates were mainly from human and animal excreta because during our sampling periods, human and animal excreta were sighted at the banks of the rivers, livestock were seen drinking water from the rivers, farmers and bricklayer were bathing and used waters from the car washing centers drained into the river. This further implicates both humans and animals as potential sources for the recovered *E. coli* pathovars. A previous study has also reported the presence of high numbers of faecal indicator bacteria originating from defective septic systems and grazing animals in freshwater sites and surface waters of developing countries [45, 46]. Other likely sources include mobilization of *E. coli* persisting in the soil [47], sediments [48] and aquatics [49].

In our study, VGs were detected in the *E. coli* isolates suggesting the presence of pathogenic *E. coli* strains in these waters. Otherwise, this may indicate an incessant input of these bacteria from a common source in the water or a combination of both. Generally, the results illustrate varied occurrence of diarrhoeagenic and non-diarrhoeagenic *E. coli* pathotypes with (91 %) of the isolates grouped under seven main *E. coli* pathotypes.

A large number of the *E. coli* isolates tested positive for toxin genes. The VG *lt* associated with ETEC strains was the most prevalent of all (45 %). This finding is worriedsome, considering the fact that it is the most common agent of traveler’s diarrhoea with food and water implicated as the modes of transmission [50, 51]. The presence of ST and/or LT enterotoxins which are commonly associated with ETEC strains have been reported by other workers in surface waters [52, 53], and are thought to originate from swine and humans with diarrhoea. ETEC strains are the most frequently isolated bacterial enteric pathogens in children below 5 years of age in developing countries and responsible for approximately 300 million diarrhoea cases and 380 000 deaths annually [54, 55], and their prevalence in surface water sources in developing countries has been documented [56]. The pathotype has been predominant followed by EPEC, EAEC and STEC in developing countries [57].

EHEC causes haemorrhagic colitis and haemolytic uremic syndrome in humans, and the key virulence factors include intimin (*eae* gene) and shiga toxins (*stx*1 and *stx*2 genes) [58]. Though, none of the isolates harboured a combination of the shiga toxin genes, nonetheless the relatively high occurrence of the *stx*1 gene (6 %) compared to *stx*2 (1 %) in the water *E. coli* isolates suggests the capability of each gene in causing acute diarrhoea in humans. This observation contradicts the relatively high occurrence of the *stx*2 gene (10 %) compared to *stx*1 (6 %) in the storm water *E. coli* isolates, which suggests that *E. coli* carrying a combination of the EHEC genes, are known to cause more severe diarrhoea in humans [15]. The most prevalent pathotypes of *E. coli* responsible for diarrhoeal diseases include enterohaemorrhagic or shiga toxin producing *E. coli* (EHEC or STEC) and enterotoxigenic *E. coli* (ETEC) [9]. The contamination of drinking or recreational waters with such *E. coli* pathotypes has been linked to waterborne disease outbreaks and mortality [53, 59].

EPEC has been shown to be a major cause of diarrhoea in young children [6]. The *eae* gene, which codes for intimin protein, was the fourth most prevalent gene in this study (6 %). This gene is necessary for intimate attachment to host epithelial cells in both the EHEC and EPEC pathotypes. Our findings tend to strongly disagree with the previous finding of significantly higher prevalence of the *eae* gene (up to 96 %) in surface water reported in other studies [22, 60]. Typical EPEC strains carry the LEE pathogenicity island, which encodes for several virulence factors, including intimin (*eae*) and the plasmid-encoded bundle forming pilus (*bfp*), which mediates adhesion to intestinal epithelial cells [34]. Therefore, all the *E. coli* isolates were further screened for the presence of the *eae* and *bfp* genes to determine their association with the EPEC pathotype. In this study, a noticeably low prevalence of the *bfp* gene (4 %) was detected, suggesting that prevalence of the EPEC-like pathotype could be expected in the surface water bodies. In addition, *eae* was also detected in 4 % isolates which lacked other typical genes from both EPEC group. This indicates prevalence of this gene in *E. coli* isolated from the freshwater environments. This finding is of great concern, as an atypical EPEC pathotype which lacks the *bfp* gene but carries the *eae* gene has been found to be a major cause of gastroenteritis worldwide [61], in patients suffering from community-acquired gastroenteritis in Melbourne, Australia [62], and from children with diarrhoea in Germany [63]. Approximately 2 % of the isolates carried both *eae* and *bfp* genes suggesting the presence of typical EPEC pathotype. The relatively low occurrence of the combination of both atypical EPEC genes in the water *E. coli* isolates is alarming due its possible significance in the cause of severe diarrhoea in humans. However, the role of atypical EPEC in diarrhoea has not been established assertively [9, 10, 64], and this study did not aim at revealing the diarrhoeagenic role of this pathotype.

In addition to the most prevalent ETEC strains obtained, EPEC and EHEC were the second- and third-more-common diarrhoeagenic pathotypes detected in this study respectively, with each group represented by 10 and 7 % isolates respectively. This is a strong indication that the three pathotypes occur widely in the surface water samples. The presence of *E. coli* strains with virulence characteristics similar to EPEC, ETEC and EHEC in fresh and estuarine waters have been previously reported [29, 30]. Generally, a few EHEC-like strains identified in this study were in agreement with other studies executed in different parts of the world [65, 66], and a low prevalence of EHEC infection has been observed in developing countries [67, 68].

EAEC is an emerging pathogen associated with diarrhoea. It has been identified in travelers, children in the developing world and human immunodeficiency virus infected patients with diarrhoea [9, 66, 69–71]. In the present study, among the DEC types, *eagg* gene of EAEC strains was the least frequently isolated adhesion VG with only 7 (2 %) strains detected in all the isolates, yet the pathotype has been an important diarrhoeagenic pathogen with its characteristic persistent diarrhoea in children and adults. This finding seems to be inconsistent with the previously reported high prevalence of the EAEC pathotype in fresh and estuarine water samples [60], but tends to align with the earlier observation of a less common DAEC, EAEC and a variety of different EHEC and EPEC pathotypes with the exception of enteroinvasive *E. coli* which was not detected in the 509 samples studied [72, 73]. Several studies have reported that contaminated food and water hygiene are the main vehicles of transmission with EAEC [50]. Similar findings have been described [74] and elsewhere [52, 75]. Our observation on the occurrences of ETEC, EPEC, EAEC and NMEC-like strains concurs with the report that ETEC is the most prevalent pathotype detected, followed by low prevalence of EPEC and NMEC, and absence of EIEC pathotypes in *E coli* isolates of surface water [76].

Uropathogenic *E. coli* (UPEC) and neonatal meningitis *E. coli* (NMEC) are the two other extra-intestinal *E. coli* (ExPEC) pathotypes that have been characterized [77, 78]. The study shows a higher prevalence of VG *pap*C (19 %)*,* associating with UPEC pathotype than the NMEC VG *ibe*A (3 %). The presence of *E. coli* strains with virulence characteristics similar to ExPEC have been reported previously in the fresh and estuarine waters [31]. This observation was of interest and may be an indication of a high potential health risk of such waters as the number of ExPEC VG(s) in *E. coli* has been suggested to be proportional to its pathogenic potential [79].

Overall, the detection of most of the VGs tested was relatively low aside from *lt* and *pap*C, ranging from 1 to 7 %. This finding correlates with the reports of [28–30], that the prevalence of *E. coli* isolates harbouring VGs in environmental waters is low ranging from 0.9 to 10 %. In the light of this, screening of a large number of isolates for possible detection of VGs is advocated. *E. coli* isolates have been concentrated from large volume (1 L) of water samples followed by an enrichment step to increase PCR detection sensitivity [80–82].

The presence of a single or multiple VGs in an *E. coli* strain does not necessarily indicate that a strain is pathogenic unless that strain has the appropriate combination of VGs to cause disease in the host [59, 83]. The pathogenic *E. coli* strains use a complex multistep mechanism of pathogenesis involving a number of virulence factors depending upon the pathotype, which consists of attachment, host cell surface modification, invasin, a variety of toxins and secretion systems which eventually lead toxins to the target host cells [9]. Thus, VGs are appropriate targets for determining the pathogenic potential of a given *E. coli* isolate [6]. The occurrence of unusual combinations of VGs in *E. coli* isolates observed in this study could be explained on the basis of horizontal gene transfer between cells, which enables the exchange of genetic material located on mobile elements (transposons, integrons or plasmids) among related or unrelated bacterial species [84]. Further screening of the *E. coli* isolates with these unusual VG patterns in tissue culture or animal models would be required to demonstrate their pathogenicity.

In this study, we collected water samples from communities with diverse human population densities and land uses to determine if these factors influence the distribution of VGs (Fig. 7). The results of this study show a relatively low and clear pattern of occurrence of VGs across the sites with a noticeable difference of occurrence of 4 VGs and 3 VGs at sites R9 and R10 respectively. Overall, it was evident that the point and non-point sources of contamination were potentially similar across the sampling sites in their characteristic features. Similarly, all the sampling sites are bordered by farm animals such as ruminants which are known to be potential sources of these VGs [11, 14, 32, 85]. Animals, humans and the environments including water sources serve as natural habitats of virulent strains of *E. coli* [6, 10, 86–88]. Storm runoffs may also increase the prevalence of microbial pathogens, including diarrhoeagenic *E. coli* pathotypes in the surface water bodies due to transport of faecal contamination from land [25].

A better understanding of the prevalence and distribution of *E. coli* pathotypes in water sources used for potable, non-potable or recreation purposes could be an important tool in the development of public health risk mitigation strategies. Pathotyping of *E. coli* isolates may also provide useful information to identify potential sources of pollution, as the principal reservoirs of ETEC and EPEC pathotypes are majorly humans and ruminants, whereas the bovine intestinal tract is the main source of the EHEC pathotype [9, 13]. The lower prevalence of the EHEC pathotype compared to other pathotypes suggests that human faecal contamination of the waterways is the main source of diarrhoeagenic *E. coli* pathotypes in the surface water as opposed to contamination from animals. This stresses the importance of controlling sources of human faecal pollution such as municipal wastewater sources, sewage leaks and overflows, wastewater treatment plant discharge to reduce potential threats to human health. The results demonstrate that the risk of contracting infection, however, may increase over time if no appropriate preventive and controlling measures are ensured. Since this study aimed at detecting *E. coli* pathotypes carrying associated VGs, it is logically reasonable to assume that actual distribution of these VGs in surface water could be relatively higher. While the ability of *E. coli* isolates described in this study to cause human diarrhoeal diseases was not established, a high proportion of isolates carrying a full set of VGs have been linked to defined pathotypes. Further screening for other VGs along with serotype testing and other assays may offer further information on pathogenicity of these isolates.

## Conclusions

Detection of *Escherichia coli* in river water in Osun State, South-western Nigeria, indicates faecal contamination and the possible presence of other enteric pathogens. The prevalence of virulence markers in *E. coli* isolates from river water sources is indicative of increased risks of mortality, especially among the vulnerable populations and immunocompromised individuals, should they contract infections through the use of river water for consumption or other household related purposes. It equally emphasizes the importance of safe water supply, good hygiene and sanitation practices both in rural and urban communities. Finally, this study has revealed a number of *E. coli* isolates positive for single and multiple VGs which indicates the presence of potential pathogenic *E. coli* in these waters and it clearly highlights the need to develop a better understanding of public health implications of occurrence of *E. coli* carrying VGs in surface waters used for potable, non-potable and recreational purposes.

## Methods

### Description of study area and collection of water samples

The ten river water sources used in this study are located in Osun State, Southwestern Nigeria. The state is an inland state with its headquarters located in Osogbo. It is bounded in the north by Kwara state, south by Ogun state, west by Oyo state and east partly by Ekiti and Ondo states. The sampling locations were coded as follows: R1: Erinle-Ede; R2: Ido-Osun; R3: Osun-Osogbo, R4: Oba-Iwo; R5: Ejigbo; R6: Ilobu-Okinni; R7: Asejire-Ikire; R8: Shasha; R9 and Ila-Oke Ila, R10: Inisha-Okuku (Fig. 1).

Water samples were collected at each site in clean, sterile 2.5 L bottles between September 2011 and August 2012 from ten sampling locations, transported to the laboratory on ice and processed within 6 h of collection. Table 1 shows the site code, location, land use and potential sources of faecal pollution of each sampling site.

**Table 1:** List of the 10 sampling sites, their location, land use and suspected sources of faecal contamination impacting each site

Site code	Location	Land use	Suspected sources of faecal pollution
R1	Erinle-Ede	Peri-urban	Tourists, coastal birds, cattle, run offs
R2	Ido Osun	Pasture	Fisher men, cattle, wild animals, run offs
R3	Osun-Osogbo	Urban	Farmers, tourists, wild animals, run offs
R4	Oba-Iwo	Peri-urban	Farmers, coastal birds, wild animals, run offs
R5	Ejigbo	Rural	Farmers and wild animals
R6	Ilobu-Okinni	Rural	Dilutions from vehicle washing centers, swimmers, animal inputs, run offs
R7	Asejire-Ikire	Peri-urban	Pollution by a bottling company, fisher men, coastal birds, wild animals
R8	Ishasha	Rural	Fisher men, dilutions from palm oil processing, swimmers, wild animals, run offs
R9	Ila-Oke Ila	Rural	Fisher men, farmers, cattle, wild animals
R10	Otin-Okuku	Pasture	Fisher men, farmers, cattle, wild animals, run offs

### Isolation of presumptive of *Escherichia coli*

The standard membrane filtration method was used for the processing and quantification of presumptive *E. coli* in the water samples [89]. A volume of 100 ml of each water sample was filtered through 0.45 mm pore size nitrocellulose membrane filters (Millipore, Ireland). The filters were placed on eosin methylene blue agar (Oxoid, England), incubated overnight at 44.5 °C for characteristic green metallic sheen colonies and thereafter counted. The isolates were further purified on *E. coli* chromogenic agar (Conda Pronadisa, Spain), incubated at 37 °C overnight and individual well-isolated typical *E. coli* colonies were selected and transferred onto nutrient agar slants for further studies. A total of 480 presumptive *E. coli* colonies were isolated during the 12-month sampling regime.

### PCR confirmation of *E. coli* and extraction of DNA

The presumptive *E. coli* were confirmed by polymerase chain reaction using the housekeeping 4-methylumbelliferyl-glucuronide (*uid*A) gene marker as previously described [90], and the positive isolates were preserved at −80 °C in 20 % glycerol. *E. coli* ATCC 25922 (ATCC, USA) was used as a positive control. The bacterial genomic DNA was extracted using the boiling method as described elsewhere [91, 92], and the recovered DNA was used as template for amplification reactions.

### PCR detection of virulence genes

Using a conventional singleplex PCR, confirmed *E. coli* isolates (*n* =300) were screened for the presence of 10 *E. coli* VGs for a number of adhesion, invasion and toxin determinants to correctly place them under the 8 pathotypes studied. The list of VGs, categorized on the basis of their functional characteristics and association with *Escherichia coli* pathotypes is shown in Table 2. The primers used for PCR detection of the VGs and other relevant characteristics are listed in Table 3. For each PCR experiment, appropriate positive and negative controls were included. The PCR amplification was performed using a thermocycler system (Bio-Rad Thermal cycler, USA). Each 25 μl PCR mixture contained 12.5 μl of PCR master mix (Thermo Scientific, (EU) Lithuania), 0.5 μl each of primer (Inqaba Biotech, SA), 5 μl of template DNA and 6.5 μl of PCR grade water. To detect the amplified product, 5 μl of amplicons was visualized by electrophoresis through a 1.8 % agarose gel (Merck, SA) at a voltage of 100 for 45 min in 0.5X TBE buffer and stained with ethidium bromide (Sigma-Aldrich, USA) using the gel documentation system (Alliance 4.7, France). Identification of the bands was established by comparison of the band sizes with molecular weight markers of 100-bp (Thermo Scientific, (EU) Lithuania). Samples were considered to be positive for a specific VG when the visible band was the same size as that of the positive control DNA. To minimize PCR contamination, DNA extraction, PCR set up, and gel electrophoresis were performed in isolated rooms. The positive controls were sourced from DSMZ Germany and included: DSM 10819 for NMEC; DSM 4819 for UPEC; DSM 8695 for EPEC; DSM 10973 for ETEC; DSM 10974 for EAEC; and DSM 10975 for EIEC except ATCC 35150 for EHEC from USA. There was no positive control available for DAEC but we went further to optimize the PCR condition of the related gene for possible detection of the expected amplicon band size.

**Table 2:** List of 10 virulence genes screened in this study, categorized based on their functional characteristics and association with *Escherichia coli* pathotypes

Pathotype	Adhesion gene	Toxin gene	Invasion gene	Function
EPEC	*eae*			Intimin/Attaching and effacing
	*bfp*			Type IV bundle-forming pili
EAEC	*eagg*			Transcriptional regulator for chromosomal gene/ Enteroaggregative adhesion
EIEC			*ipa*H	Invasion plasmid antigen
ETEC		*lt*		Heat-labile toxin
DAEC	*daa*E			
EHEC		*stx*1		Shiga-toxin 1
		*stx*2		Shiga-toxin 2
UPEC	*pap*C			P fimbriae chaperone
NMEC			*ibe*A	Invasion of brain endothelium

**Table 3:** Primer sequences, expected amplicon sizes and their cycling conditions

Target strain	Target gene	Primer sequence (5′ → 3′)	Amplicon size (bp)	PCR cycling condition	Reference
*E. coli*	*uid*A	F: AAA ACG GCA AGA AAA AGC AG	147	5 min initial denaturation at 94 °C followed by 35 cycles of 95 °C for 30 s, 58 °C for 1 min, 72 °C for 1 min and final extension at 72 °C for 8 min	[90]
		R: ACG CGT GGT TAA CAG TCT TGC G			
EPEC	*eaeEae*	F: TCA ATG CAG TTC CGT TAT CAG TT	482	15 min initial denaturation at 95 °C followed by 35 cycles of 94 °C for 45 s, 55 °C for 45 s, 68 °C for 2 min and final extension at 72 °C for 5 min	[93]
		R: GTA AAG TCC GTT ACC CCA ACC TG			
	*bfpBfp*	F: GGA AGT CAA ATT CAT GGG GGT AT	300	2 min initial denaturation at 94 °C followed by 40 cycles of 94 °C for 1 min, 55 °C for 1 min, 72 °C for 1 min and final extension at 72 °C for 5 min	[93]
		R: GGA ATC AGA CGC AGA CTG GTA GT			
ETEC	*ltLt*	F: GGC GAC AGA TTA TAC CGT GC	450	2 min initial denaturation at 94 °C followed by 35 cycles of 94 °C for 1 min, 55 °C for 1 min, 72 °C for 1 min and final extension at 72 °C for 5 min	[94]
		G: CGG TCT CTA TAT TCC CTG TT			
EAEC	*eaggEagg*	F: AGA CTC TGG CGA AAG ACT GTA TC	194	15 min initial denaturation at 95 °C followed by 35 cycles of 94 °C for 45 s, 55 °C for 45 s, 68 °C for 2 min and final extension at 72 °C for 5 min	[95]
		R: ATG GCT GTC TGT AAT AGA TGA GAA C			
EIEC	*ipa*H	F: CTC GGC ACG TTT TAA TAG TCT GG	933	2 min initial denaturation at 94 °C followed by 35 cycles of 94 °C for 1 min, 55 °C for 1 min, 72 °C for 1 min and final extension at 72 °C for 5 min	[96]
		R: GTG GAG AGC TGA AGT TTC TCT GC			
DAEC	*daa*E	F: GAA CGT TGG TTA ATG TGG GGT AA	542	2 min initial denaturation at 94 °C followed by 40 cycles of 92 °C for 30 s min, 59 °C for 30 s, 72 °C for 30 s and final extension at 72 °C for 5 min	[96]
		R: TAT TCA CCG GTC GGT TAT CAG T			
EHEC	*stx*1	F: CAG TTA ATG TGG TGG CGA AGG	384	15 min initial denaturation at 95 °C followed by 35 cycles of 94 °C for 45 s, 55 °C for 45 s, 68 °C for 2 min and final extension at 72 °C for 5 min	[72]
		R: CAC CAG ACA ATG TAA CCG CTG			
	*stx*2	F: ATC CTA TTC CCG GGA GTT TAC G	584	2 min initial denaturation at 94 °C followed by 35 cycles of 94 °C for 1 min, 55 °C for 1 min, 72 °C for 1 min and final extension at 72 °C for 5 min	[72]
		R GCG TCA TCG TAT ACA CAG GAG C			
NMEC	*ibe*A	F: TGG AAC CCC GCT CGT AAT ATA C	342	2 min initial denaturation at 94 °C followed by 30 cycles of 94 °C for 1 min, 55 °C for 1 min, 72 °C for 1 min and final extension at 72 °C for 5 min	[96]
		R: CTG CCT GTT CAA GCA TTG CA			
UPEC	*pap*C	F: GAC GGC TGT ACT GCA GGG TGT GGC G	328	2 min initial denaturation at 94 °C followed by 30 cycles of 94 °C for 1 min, 55 °C for 1 min, 72 °C for 1 min and final extension at 72 °C for 5 min	[97]
		R: ATA TCC TTT CTG CAG GGA TGC AAT A			

### Statistical analysis

Statistical analysis was performed using IBM Statistical Package for Social Sciences [(SPSS) Version 20 software]. Data on *E. coli* mean counts for each site were recorded in 100 ml per CFU. The one way analysis of variance (ANOVA) was performed to investigate the existence of any correlation between *E. coli* counts, the difference in VG distribution and the degree of correlation between the number of *E. coli* isolates and the number of VGs observed with respect to each site. Correlations and test of significance were considered statistically significant when *P* values were < 0.05.

## Abbreviations

ANOVA: Analysis of Variance
APEC: Avian Pathogenic *Escherichia coli*
ATCC: American Type Culture Collection
CDEC: Cell-Detaching *Escherichia coli*
CFU: Colony Forming Unit
DAEC: Diffusely Adherent *Escherichia coli*
DEC: Diarrhoeagenic *Escherichia coli*
DNA: Deoxyribonucleic Acid
EAEC: Enteroaggregative *Escherichia coli*
EHEC: Enterohemorrhagic *Escherichia coli*
EIEC: Enteroinvasive *Escherichia coli*
EPEC: Enteropathogenic *Escherichia coli*
ETEC: Enterotoxigenic *Escherichia coli*
ExPEC: Extra-Intestinal Pathogenic *Escherichia coli*
InPEC: Intestinal Pathogenic *Escherichia coli*
NMEC: Neonatal Meningitis *Escherichia coli*
PCR: Polymerase Chain Reaction
R: River
SAMRC: South African Medical Research Council
SPSS: Statistical Package for Social Sciences
UPEC: Uropathogenic *Escherichia coli*
VG: Virulence Gene
WRC: Water Research Commission
